# New Appliances and Things Medical

**Published:** 1895-01-19

**Authors:** 


### NEW APPLIANCES AND THINGS MEDICAL.

[We shall be glad to receive, at our Office, 428, Strand, London, W.C., from the manufacturers, specimens of all new preparations and drugs which may be brought out from time to time.]

### COCOA AND FANCY CHOCOLATES.

(Ph: SUCHARD (NEUCHÂTEL) 36, HOLBORN VIADUCT, E.C.)

Suchard's chocolate is known to everybody, and one and all are agreed as to the excellence of this particular class of specialities. We have before us, however, a sample of their soluble cocoa, a product much less widely known, but by no means less worthy of notice. This cocoa is largely freed from fatty material, possesses in a marked degree the nutty flavour peculiar to the raw product, is prepared in the form of a fine powder, and is readily soluble in water; in fact, it possesses in a marked degree those qualities which are most esteemed by connoisseurs of cocoa. Persons of weak digestive powers are only just beginning to appreciate the value of cocoa as a substitute for tea, with its concomitants, tannin and allied substances. It is a knowledge, however, that once gained is never forgotten, and which is bound to grow, conducing to a greatly increased consumption of cocoa. Suchard's cocoa is assured of a honourable place amongst the cocoas of the future.

### "TOE POST" SHOES AND DIGITATED HOSE.

(HOLDEN BROTHERS, 223½, REGENT STREET, W., LONDON.)

This new device of Holden Brothers, for maintaining the big toe in place, is a most ingenious invention. The shoes, which are intended for the most part for children, contain, as it were, a separate division or stall for the big toe, which is thus not only separated from its fellows, but is also kept in its natural position. The natural position for the hallux to assume is one in a straight line with the corresponding metatarsal bone of the foot. As a matter of fact, in an adult one never comes across a foot in which the big toe is in its normal position; it always inclines at a greater or less angle towards the outer side of the foot. The metatarso-phalangeal joint of the big toe becomes an angular projection on the inner side of the foot, and is thus subjected to undue pressure; in many cases this leads directly to a bunion, a most unenviable and intractable condition. If the great toe is kept in its proper position from childhood onwards, a bunion at the metatarso-phalangeal joint of the big toe is almost an impossibility. We believe it to be an equal impossibility to have an outcurved big toe, if Toe Post shoes are systematically worn. And this is no hardship, since the shoes are decidedly comfortable when worn with a digitated stocking. Digitated hose, adapted for wear along with the above shoes, can be procured at the same establishment.

### CHIVERS' JELLIES.

(MESSRS. CHIVERS AND SONS, HISTON, CAMBRIDGE.)

We have made trial of a few samples of the above jellies. We find that they are prepared from pure gelatine, and flavoured with a variety of aromatic fruit syrups. They will stand a considerable amount of dilution, and yet set firmly and easily; in other words, they are a decidedly economial variety of jelly. We strongly recommend them for invalids, since their delicacy of flavour will be highly appreciated by those who can only be tempted by the most dainty of foods. We were glad to bring them to the notice of a lady who had given up the use of ready-made jelly, as she had had one or two dangerous experiences of broken glass finding its way into jellies taken from glass receptacles.

### GLYCOLA SPECIALITIES.

(MESSRS. T. CLARK, CROUCH END, N.)

We have received specimens of Messrs. T. Clark's glycola specialities, consisting of soap, toilet cream, and tooth powder. All are excellent of their kind, and most agreeable in use. The soap is stated to be free from potash and soda, and is superfatted and well milled, so that it is economical and pleasant for the toilet. We especially commend the perfume of lavender used to scent this soap; it is altogether superior to that generally to be procured, and is sold at a moderate price. Glycola tooth powder is antiseptic and effective. The carbolic used in its preparation is hardly perceptible, and the powder is very pleasant. The glycola cream is justly stated to be neither sticky nor greasy, and thus is much more agreeable in application than either glycerine or cold cream, for both of which it is a substitute, and is intended to fulfil the same purposes. The only point not in its favour is that its form is much more liquid than either of its rivals, and it is therefore liable to be more extravagantly used. This is but a small drawback, and will not, we feel sure, interfere with the popularity which Messrs. Clark's manufactures are likely to attain.

